# Supplementary material for: Mechanisms governing the pioneering and redistribution capabilities of the non-classical pioneer PU.1
Source: Nat Commun. 2020 Jan 21;11:402. doi: 10.1038/s41467-019-13960-2 (PMC6972792; doi:10.1038/s41467-019-13960-2)
Supplement: Supplementary file 7 — Source data [file 41467_2019_13960_MOESM7_ESM.zip › Source_Data/FigureS2/FigureS2B_MotifScanOutput/KmeansCluster7/homerResults/motif31.similar.html]

motif31

## Information for motif31

C
A
T
G
G
T
A
C
A
G
C
T
C
T
G
A
A
G
T
C
T
C
G
A
A
G
T
C
C
G
T
A
A
T
G
C
G
A
C
T
C
A
G
T
  
Reverse Opposite:  

G
T
C
A
C
G
T
A
T
A
C
G
C
G
A
T
C
T
A
G
A
G
C
T
C
T
A
G
A
G
C
T
C
T
G
A
A
C
T
G
G
T
A
C
  

|  |  |
| --- | --- |
| p-value: | 1e-41 |
| log p-value: | -9.552e+01 |
| Information Content per bp: | 1.896 |
| Number of Target Sequences with motif | 68.0 |
| Percentage of Target Sequences with motif | 2.12% |
| Number of Background Sequences with motif | 69.6 |
| Percentage of Background Sequences with motif | 0.16% |
| Average Position of motif in Targets | 102.3 +/- 31.2bp |
| Average Position of motif in Background | 108.2 +/- 62.6bp |
| Strand Bias (log2 ratio + to - strand density) | 0.2 |
| Multiplicity (# of sites on avg that occur together) | 1.00 |
| Motif File: | file (matrix) reverse opposite |

### Similar de novo motifs found

|  |  |  |  |  |  |  |  |
| --- | --- | --- | --- | --- | --- | --- | --- |
| Rank | Match Score | Redundant Motif | P-value | log P-value | % of Targets | % of Background | Motif file |
| 1 | 0.851 | A G T C G A C T C T G A A T G C C T G A A T G C G T C A T A C G A C G T A C G T | 1e-38 | -88.980997 | 2.99% | 0.46% | motif file (matrix) |
| 2 | 0.778 | A C T G A C G T A C T G A C G T A C T G A G C T C T A G A C T G A G T C | 1e-27 | -62.269003 | 4.42% | 1.43% | motif file (matrix) |
| 3 | 0.889 | A C G T C G T A A G T C C T G A A G T C C G T A A T G C A C G T A C G T | 1e-26 | -59.932209 | 2.02% | 0.32% | motif file (matrix) |
| 4 | 0.849 | A C T G A G T C A G C T C G T A A G T C G T C A A G T C C G T A | 1e-16 | -38.333156 | 4.45% | 1.94% | motif file (matrix) |
